# Low Concentration of Aluminum-Stimulated Pollen Tube Growth of Apples (*Malus domestica*)

**DOI:** 10.3390/plants11131705

**Published:** 2022-06-28

**Authors:** Chen Zhang, Pengxue Xie, Qing Zhang, Yu Xing, Qingqin Cao, Ling Qin, Kefeng Fang

**Affiliations:** 1 Beijing Advanced Innovation Center for Tree Breeding by Molecular Design, Beijing University of Agriculture, Beijing 102206, China; 202030512018@bua.edu.cn (C.Z.); xingyu@bua.edu.cn (Y.X.); caoqingqin@bua.edu.cn (Q.C.); 2 College of Landscape Architecture, Beijing University of Agriculture, Beijing 102206, China; 3 Key Laboratory for Agricultural Application and New Technique, College of Plant Science and Technology, Beijing University of Agriculture, Beijing 102206, China; 201530221214@bua.edu.cn (P.X.); zhangqing@bua.edu.cn (Q.Z.)

**Keywords:** *Malus domestica*, Aluminum, calcium, actin, cell wall components, pollen tube

## Abstract

Aluminum (Al) is an important element in soil constitution. Previous studies have shown that high concentration of Al affects the normal growth of crops, resulting in crop yield reduction and inferior quality. Nevertheless, Al has also been referred to as a beneficial element, especially when used at low concentrations, but the cytological mechanism is not clear. Influences of low concentration AlCl_3_ on the pollen tube growth of apple (*Malus domestica*) and its possible cytological mechanism were investigated in this study. The results showed that 20 μM AlCl_3_ promoted pollen germination and tube elongation; 20 μM AlCl_3_ enhanced Ca^2+^ influx but did not affect [Ca^2+^]c of the pollen tube tip; and 20 μM AlCl_3_ decreased acid pectins in pollen tubes but increased esterified pectins and arabinan pectins in pollen tubes. According to the information provided in this research, 20 μM AlCl_3_ stimulated growth of pollen tubes by enhancing Ca^2+^ influx and changing cell wall components.

## 1. Introduction

Aluminum (Al) is the most abundant and widely distributed metallic element in the earth’s crust [1]. In acidic soil conditions (pH < 5), the conjugated Al dissociates into toxic active Al, which is one of the important factors restricting crop growth [2]. Nevertheless, Al has also been referred to as a beneficial element, especially when used at low concentrations. Osaki et al. (1997) reported that low concentration of Al (3 mg/L) stimulated plant growth and enhanced P uptake [3]. Similar responses have also been reported by Watanabe et al. (2005) in Indian rhododendron [4]. Low concentration of Al promoted root and shoot growth and antioxidant activity of common bean [5] and the leaf growth of maize [6]. Pilon-Smits et al. (2009) reported that the beneficial influences of Al in plants were relevant to the ability to promote the absorption of K^+^ and Mg^2+^, increase P utilization and reduce Fe toxicity, thus accelerating their vegetative growth in plants [7]. In root culture, adding Al could promote the growth of primary and lateral roots, increase the activity of antioxidant enzymes and delay aging [7]. Therefore, Al could be used as a biological stimulant to promote crop growth and productivity, especially when used at low concentrations [7].

Pollen tube is the fast-growing male gametophyte of angiosperms in nature, its elongation shows a typical polarized growth pattern similar to that of fungal hyphae, root hairs, and neuronal axon guidance [8]. Pollen tubes grow far away and transport male gametes to the embryo sac, where double fertilization happens [9]. The rapid growth of the pollen tube is highly dependent on the precise and orderly material transport of the cell membrane, cell wall, functional proteins, and other necessary materials to the tip growth point [9]. The rapid movement of organelles and vesicles depends on the dynamics of the cytoskeleton, which is constantly reorganized in response to external signals [10,11]. In angiosperms, the wall of the pollen tube consists of two layers; the outer layer is dominated by cellulose and pectin, and the inner layer is callose [12], which is different from those of somatic cells [13,14].

Ca^2+^ plays essential roles in a range of plant growth and development processes such as polarity formation, growth, cell division, cell wall formation, and the transduction and regulation of internal and external signals [15]. There exists a cytosolic Ca^2+^ concentration ([Ca^2+^]c) gradient at pollen tube tip, which is mainly maintained by extracellular Ca^2+^ influx [15]. The [Ca^2+^]c gradient is critical for pollen-tube-tip growth [1].

Pollen tube growth is regulated by Ca^2+^ and is sensitive to Al [16]. Pollen tubes are an excellent standard system for studying the effects of drugs and pollutants [16]. Therefore, the pollen tube provides a useful system through which to study the cytological mechanism of low Al^3+^ concentration on pollen tube growth.

Previous studies have shown that high concentrations of Al affected the normal growth of pollen tubes [17]. High concentrations of Al inhibited geraldton waxflower pollen germination through inhibition of Ca^2+^ influx into the pollen grains and caused a rapid tip bursting [18]. High concentrations of Al interfered with the pectin–calcium binding sites and caused a decrease in geraldton waxflower cell wall elasticity [18]. High concentrations of Al caused cell wall thickening at the tip of *Lily* pollen tubes, and the tube diameter increased abnormally [19]. High concentrations of Al inhibited apple pollen germination and tube growth, decreased pollen tube apex calcium influx, disrupted the [Ca^2+^]c gradient, altered actin filament orientation, and affected the accumulation and distribution of callose, acid pectins, esterified pectins, and arabinogalactan proteins [1].

Nevertheless, relatively few researches have been conducted on the beneficial effects of Al on pollen tubes [20]. Hiromi et al. (1997) reported that 3 μM Al^3+^ stimulated the growth of tea pollen tube [21], but the cytological mechanism is not clear. The effects of a low concentration of Al on apple pollen tube growth is not clear. Thus, the cytological mechanism of low Al^3+^ concentration on pollen tube growth was studied in the present study.

## 2. Results

### 2.1. 20 µM AlCl_3_ Affected M. domestica Pollen Germination and Pollen Tube Growth

In our research, to evaluate the effect of Al^3+^ on pollen germination and tube growth, different concentrations of Al^3+^, including 0, 10, 20, 30, 50, and 100 µM, were added to the medium. The results showed that 20 µM AlCl_3_ promoted pollen germination, and 20–100 µM AlCl_3_ was benefit to pollen tube elongation (Table 1). Low concentrations of Al could promote pollen germination. The germination rate of the control pollen tubes was 35.09% (Table 1). The germination rate of the 10 µM AlCl_3_ treated pollen tubes was 42.07% (Table 1). However, 20 µM AlCl_3_ increased the germination rate to 57.25% (Table 1). On the other hand, 30 µM AlCl_3_ decreased the germination rate to 42.88% (Table 1). In addition, 50 µM AlCl3 decreased the germination rate to 33.89% (Table 1), and 20 µM AlCl_3_ worked best. Our results showed that 20 μM AlCl_3_ stimulated pollen germination.

Low concentrations of Al could promote pollen tube growth. After 2 h incubation, the length of control pollen tube was 101.83 µm (Table 1). The length of pollen tubes was 142.36 µm when treated with 20 µM AlCl_3_ (Table 1). The length of pollen tubes was 144.64 µm when treated with 50 µM AlCl_3_ (Table 1). There was no significant difference between the 50 µM and 20 µM AlCl_3_ treatments. Our results show that 20 μM and 50 µM AlCl_3_ promoted pollen tube elongation.

**Figure 1:**
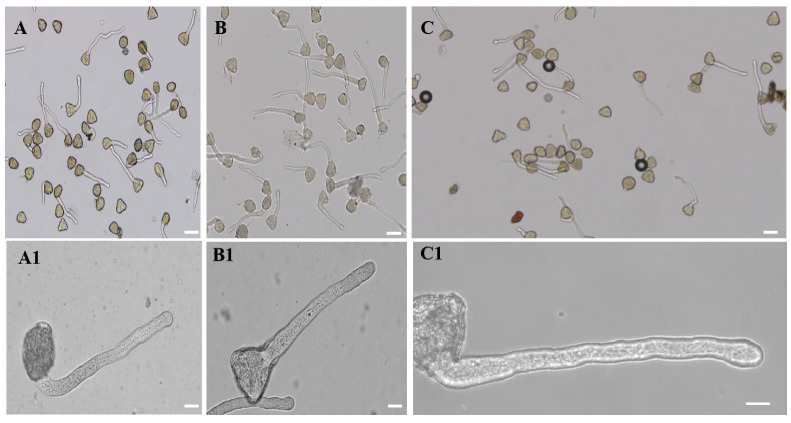
Effect of different concentration of AlCl_3_ on pollen tube morphology. Control pollen tubes. (**A**) Control pollen tubes. (**A1**) One control pollen tube. (**B**) Pollen tubes treated with 20 µM AlCl_3_. (**B1**) One pollen tube treated with 20 µM AlCl_3_. (**C**) Pollen tubes treated with 50 µM AlCl_3_. (**C1**) One pollen tube treated with 50 µM AlCl_3_. Bar = 25 µm.

The morphology of pollen tubes was observed under a microscope, and it was found that the control, 20-, and 50-μM-AlCl_3_-treated pollen tubes were regular (Figure 1A, A1 and B, B1). Based on these results, 20 μM AlCl_3_ was used in the following experiments as the Al treatment; the absence of Al (AlCl_3_) in the incubation medium was used as control.

### 2.2. Effect of 20 µM AlCl_3_ on Ca^2+^ Flux and [Ca^2+^]c in Pollen Tubes of M. domestica

**Figure 2:**
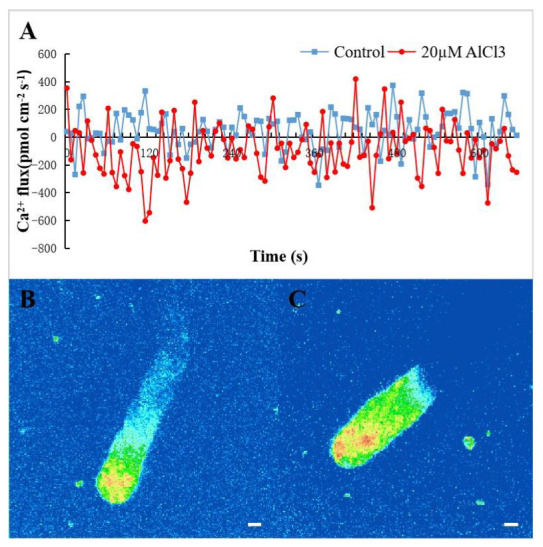
Effects of 20 µM AlCl_3_ on Ca^2+^ flux and [Ca^2+^]c in pollen tubes of *M. domestica.* (**A**) Ca^2+^ flux of *M. domestica* pollen tube apex under different condition. The blue line stands for Ca^2+^ flux of control pollen tube, while red line represents Ca^2+^ flux of pollen tubes treated with 20 μM. (**B**) The [Ca^2+^]c at the tip of the control pollen tube labeled with Fluo-3/AM ester under CLSM. (**C**) The [Ca^2+^]c at the tip of the pollen tube treated with 20 μM AlCl_3_. Bar = 10 µm. Note: The negative value of Ca^2+^ flux represents absorption (influx), and the positive value represents efflux.

Ca^2+^ influx and efflux were generally equal at the control tube tip after a 2 h culture (Figure 2 blue line). In Figure 2, negative values represent Ca^2+^ influx, and positive values represent Ca^2+^ efflux. After statistical analysis, there were more amplitudes below the x-axis than above the x-axis, so the result could be drawn that the influx of Ca^2+^ increased at the tip of the pollen tube treated with 20 µM AlCl_3_ (Figure 2 red line). The results indicated that 20 µM AlCl_3_ could promote the absorption of Ca^2+^ at the tip of the pollen tube. A representative [Ca^2+^]c gradient was present within 20–30 μm of control and 20 μM AlCl_3_ treated pollen tube tip (Figure 2B,C). The results showed that 20 μM AlCl_3_ did not affect [Ca^2+^]c gradient.

### 2.3. Effect of 20 µM AlCl_3_ on Actin Filaments in Pollen Tubes of M. domestica

**Figure 3:**
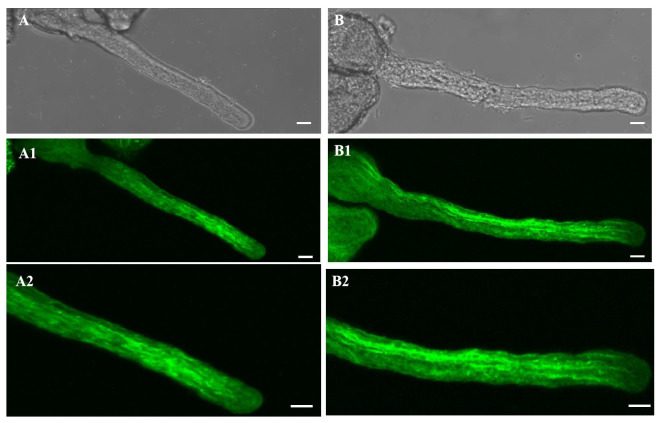
Effect of 20 µM AlCl_3_ on actin filaments of pollen tubes of *M. domestica.* (**A**) Bright-field image of a control pollen tube. (**A1**) Actin filaments of the pollen tube in A, labeled with 0.2 μM phalloidin-FITC under CLSM. (**A2**) Larger magnification of **A1**. (**B**) Bright-field image of a pollen tube treated with 20 µM AlCl_3_. (**B1**) Actin filaments of the pollen tube in B. (**B2**) Larger magnification of **B1**. Bar = 10 µm.

In both the control and the 20-µM-AlCl_3_-treated pollen tubes, the actin filaments were arranged parallel to the growth direction of the pollen tubes and connected with the actin filaments in the pollen grain (Figure 3A,A1). The 20 µM AlCl_3_ had no obvious effect on the actin filaments (Figure 3B,B1).

### 2.4. Effect of 20 µM AlCl_3_ on Distribution of Cellulose and Callose in Pollen Tube Cell Wall of M. domestica

**Figure 4:**
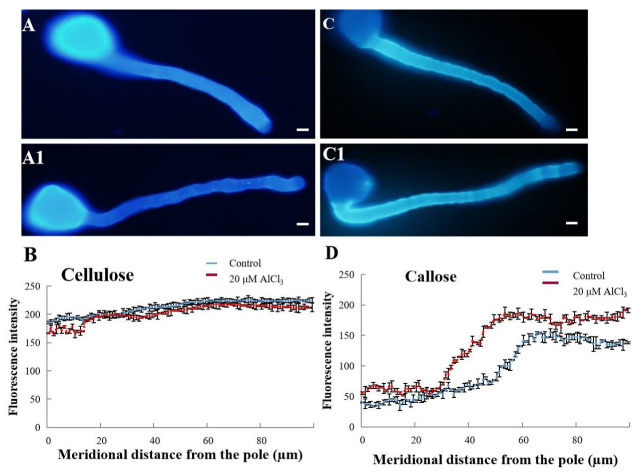
Effect of 20 µM AlCl_3_ on cellulose and callose of *M. domestica* pollen tubes. (**A**) Cellulose distribution in control pollen tube labeled with Calcofluor White Stain under fluorescence microscope. (**A1**) Cellulose of pollen tube treated with 20 µM AlCl_3_. (**B**) Quantitative analysis of the fluorescent intensity of cellulose in the wall of control pollen tubes (blue line) and 20 μM AlCl_3_ treated pollen tubes (red line). (**C**) Callose distribution in control pollen tube labeled with aniline blue under fluorescence microscope. (**C1**) Callose of pollen tube treated with 20 µM AlCl_3_. (**D**) Quantitative analysis of the florescent intensity of callose in the wall of control (blue line) and 20 μM AlCl_3_ treated pollen tubes (red line). Arrows indicate the tips of pollen tubes. Bar = 10 µm.

Cellulose was evenly distributed on the wall of the control pollen tube, and the fluorescent intensity of the tip was slightly weaker (Figure 4A). The distribution pattern of cellulose in the pollen tube treated with 20 µM AlCl_3_ changed little (Figure 4A1), indicating that 20 µM AlCl_3_ had little influence on the distribution of cellulose in pollen tubes. The result was further supported by quantitative analysis (Figure 4B). In the control and 20-µM-AlCl_3_-treated pollen tubes, callose decreased gradually from the distal region toward the tip with no fluorescent signal at the tube tip (Figure 4C,C1). The result was further supported by quantitative analysis (Figure 4D).

### 2.5. Effects of 20 µM AlCl_3_ on Distribution of Pectins in Pollen Tube Cell Wall of M. domestica

**Figure 5:**
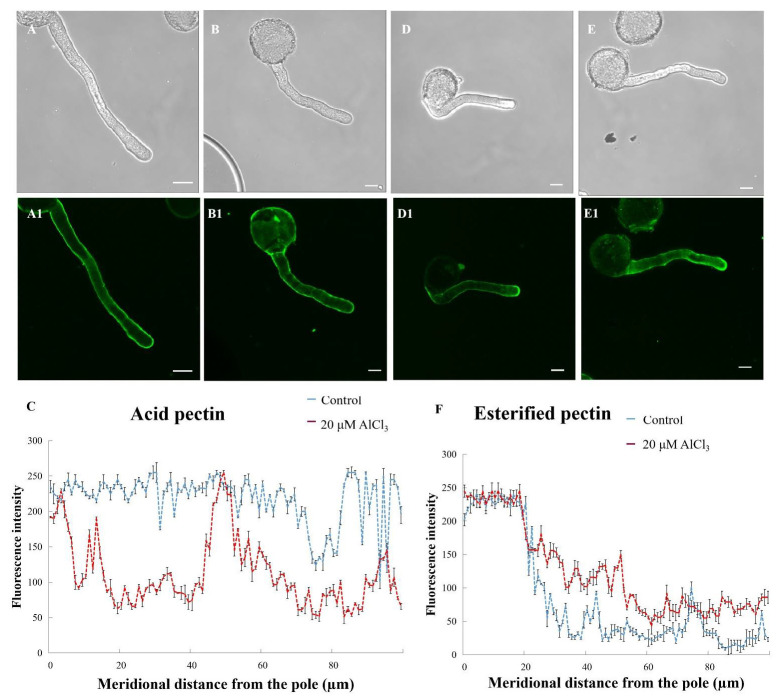
Effect of 20 µM AlCl_3_ on pectin distribution of pollen tube wall of *M. domestica.* (**A**) Bright-field image of a control pollen tube. (**A1**) Acid pectins of the control pollen tube in A labeled with JIM 5 under CLSM. (**B**) Bright-field image of a pollen tube treated with 20 µM AlCl_3._ (**B1**) Acid pectins of the pollen tube in B. (**C**) Quantitative analysis of the fluorescent intensity of acid pectins (JIM 5) in the wall of control (blue line) and 20 µM AlCl_3_ (red line) pollen tubes. (**D**) Bright-field image of a control pollen tube. (**D1**) Esterified pectins of the pollen tube in D labeled with JIM 7 under CLSM. (**E**) Bright-field image of a pollen tube treated with 20 µM AlCl_3._ (**E1**) Esterified pectins of the pollen tube in E. (**F**) Quantitative analysis of the fluorescent intensity of esterified pectins of control (blue line) and 20 µM AlCl_3_ treated (red line) pollen tubes. Bar = 10 µm.

Acid pectins (JIM 5) was evenly distributed on the control pollen tube wall (Figure 5A1). At the tip of pollen tube wall, the fluorescent intensity of the 20-µM-AlCl_3_-treated pollen tubes became weaker, indicating that 20 µM AlCl_3_ decreased acid pectins (Figure 5B1,C). Esterified pectins (JIM 7) was distributed on both the control and 20-µM-AlCl_3_-treated pollen tube wall with strong fluorescence at the tip (Figure 5E1). Compared with the control pollen tube, stronger fluorescence was observed at the tip of the 20 μM AlCl_3_ pollen tube (Figure 5F). Thus, 20 μM AlCl_3_ increased esterified pectins in the pollen tubes.

**Figure 6:**
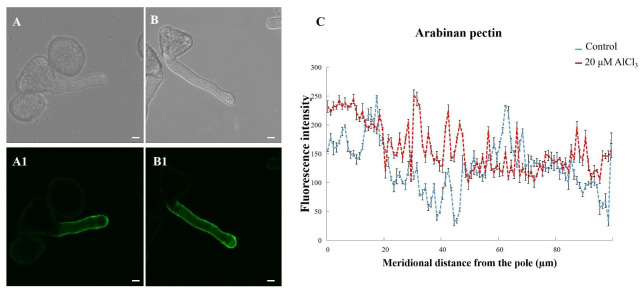
Effect of 20 µM AlCl_3_ on the distribution of arabinan pectin in pollen tube wall of *M. domestica.* (**A**) Bright-field image of a control pollen tube. (**A1**) Arabinan pectin of the pollen tube in A labeled with LM 6 under CLSM. (**B**) Bright-field image of a pollen tube treated with 20 μM AlCl_3._ (**B1**) Arabinan pectin of the pollen tube in B. (**C**) Quantitative analysis of the fluorescent intensity of control (blue line) and 20 µM AlCl_3_ treated (red line) pollen tubes. Bar = 10 µm.

LM6 could label (1→5)-α-L-arabinan, a structural feature of the side chains of pectins [22]. To specify how much pectin epitopes are involved in pollen tube growth, the antibody (LM 6) was used to label arabinan pectins. Arabinan pectins was distributed on both the control and the 20-µM-AlCl_3_-treated pollen tube walls. At the tip of the pollen tube, stronger fluorescence was observed on the 20-μM-AlCl_3_-treated pollen tube (Figure 6B1). Thus, 20 μM AlCl_3_ increased arabinan pectins in pollen tubes.

### 2.6. Effects of 20 µM AlCl_3_ on Chemical Composition of Pollen Tube Cell Wall of M. domestica

**Figure 7:**
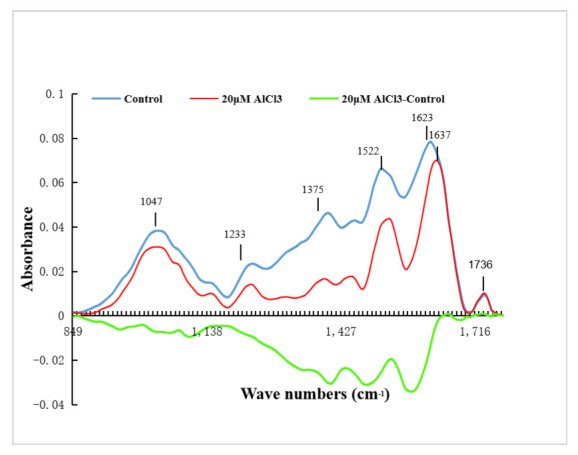
FTIR spectra from the tip of pollen tubes of *M. domestica.* FTIR spectra from the tip regions of control pollen tubes (**blue line**), 20 µM AlCl_3_ treated pollen tubes (**red line**), and the FTIR differential spectrum generated by digital subtraction of the red spectra from blue spectra (**green line**).

The infrared spectrograms of the pollen tube tips were analyzed. The difference spectrum was made, and the results showed that the spectrum of pollen tube tip changed after 20 µM AlCl_3_ treatment (Figure 7). In the spectrum of the cell wall at the tip of the control pollen tubes, the absorption peak of the saturated ester was 1736 cm^−1^, while 1623 cm^−1^ and 1522 cm^−1^ were the absorption peaks of amide I and amide II, respectively; 1455 cm^−1^ was the absorption peak of carboxylic acid. The range of 1200–900 cm^−1^ was the absorption peak of carbohydrate. It could be seen from the Figure 7 that the absorption peak of saturated ester rose slightly, which indicated that the esterified pectins in the cell wall at the tip of 20 µM AlCl_3_ treated pollen tubes increased. The absorption peak of carboxylic acid decreased, indicating that acid pectins in the tip cell wall of 20 µM AlCl_3_ treated pollen tube decreased. The absorption peak intensity of amide I band and amide II band decreased, and the absorption peak position changed, indicating the protein composition and content in the cell wall changed. The intensity of the carbohydrate absorption peak increased. These results were similar to those of the above-mentioned immunofluorescence labeling.

## 3. Discussion

Al is the most abundant metal element in the earth’s crust, accounting for about seven percent of the total mineral content of the soil, and the toxicity of Al is a serious problem for agricultural plants [23]. Nevertheless, Al has also been considered to be a beneficial element for some plant species, especially when used at low concentrations [24]. A previous study reported that low concentrations of Al could promote tea pollen germination and pollen tube growth [21].

In angiosperms, pollen tubes transport two sperm cells to the egg cell and central cell in embryo sac for double fertilization, which is of great significance to sexual reproduction [17]. In this study, low concentration Al^3+^ stimulated pollen germination and tube growth.

### 3.1. Relationship among Al, Ca^2+^ Flux and [Ca^2+^]c

Ca^2+^ is necessary for pollen tube growth, and its function has been proved by many studies. Ca^2+^ is involved in the early stages of pollen germination and pollen tube growth [25]. Firstly, [Ca^2+^]c accumulates in pollen germination apertures rapidly after hydration [25]. If [Ca^2+^]c is not established in the pollen grains, there is no protuberance, and germination is inhibited [25]. Secondly, Ca^2+^ accumulates at the tip of pollen tube when pollen tube elongated [25]. Destruction or modification of the [Ca^2+^]c gradient at the tip will interrupt the growth of the pollen tube [26]. It has been reported that pollen tubes require external Ca^2+^ concentrations between 10 μM and 10 mM [27]. In rice, maize, and wheat roots, 20 μM Al^3+^ treatment reduces Ca^2+^ absorption [28,29]. In the *arabidopsis* root, Very and Davies (2000) suggested that Al’s inhibition (100 μM) of Ca^2+^ absorption might be related to Al’s inhibition of Ca^2+^ channels [30]. Rengel (1992) demonstrated that Al (≥100 μM) could specifically bind to calcium channels, thereby competitively inhibiting Ca^2+^ absorption in wheat roots [31]. Ca^2+^ uptake was inhibited only under high concentration of Al treatment (≥100 μM) [31]. Fang et al. (2020) suggested that 600 μM AlCl_3_ inhibited Ca^2+^ influx and disturbed the [Ca^2+^]c gradient, leading to the inhibition of pollen tube growth [1]. Ca^2+^ plays a key role in the mechanism of resistance against Al [17]. Hepler (2005) reported that there were internal stores (endoplasmic reticulum, vacuole, and mitochondria) where stored and released Ca^2+^ to maintain local gradients [32]. In our study, the results showed that 20 μM AlCl_3_ enhanced Ca^2+^ influx but did not affect the [Ca^2+^]c of the pollen tube tip. We speculated that excess Ca^2+^ might store in the endoplasmic reticulum, vacuole, and mitochondria.

### 3.2. Al Altered the Deposition of Pollen Tube Wall Components

Cell walls are highly complex structures, and the main components of plant cell walls are polysaccharide [33]. The components of cell wall are not constant but will adjust with the changes of external environmental conditions and become a barrier to resist adverse external environment [34]. It has been shown that the change of cell wall components plays an important role in resisting Al toxicity [35]. The cell wall of the root tip is the first place to contact and feel Al^3+^ and is the first barrier of cells to resist Al toxicity [33]. In this study, Calcofluor white was used to detect cellulose on apple pollen tube wall. In addition, the distribution of cellulose in the pollen tube was not affected by 20 µM AlCl_3_.

Callose plays an important role in a series of processes of plant development and in the resistance to biological and abiotic stresses. One symptom of root cells exposed to Al stress is callose formation [17]. Callose formation can be regarded as a parameter of Al sensitivity and is positively correlated with pectin content [17]. It has been reported that, on one hand, the rapid synthesis or degradation of callose in plants participates in the growth and metabolism of plants [36]; on the other hand, callose deposition can also serve as a physical barrier to restrict or prevent pathogens and thus resist the invasion of pathogens [37]. In flowering plants, callose is the main component of functional substances in multiple stages of pollen tube development [38]. Callose is also the main component of pollen tubes and is formed with pollen tube lengthening and usually found mainly at the distal region, without callose at the pollen tip [39]. Fang et al. (2020) suggested that callose accumulated at the tips of pollen tubes treated with 600 μM AlCl_3_, indicating Al toxicity influenced the tube growth by disturbing callose distribution at the apple pollen tube tip [1]. In this study, callose was mainly distributed on both sides of the control pollen tube wall, and almost no callose was observed at the tip. The 20 µM AlCl_3_ did not affect callose distribution.

Pectin is a galacturonic acid rich branch heteropoly, which is the most complex polymer in a cell wall. Pectin mainly controls cell-wall porosity and intercellular adhesion and plays an important role in pollen tube growth, pathogen resistance, and cell swelling [40]. Nagayama et al. (2019) found that there was a significant correlation between the pectin content in rice cell wall and rice’s ability to tolerate Al^3+^ [41]. When the pectin content increased, rice’s ability to tolerate Al^3+^ increased [41]. A large number of studies have shown that Al tolerance in plants is related to the pectin content in the root cell wall and the degree of pectin methylation [42]. Esterified pectin residues are produced in the Golgi apparatus and released at the tip of the pollen tube, which cause the tube to expand [43,44]. At the pollen tube wall behind the apex, esterified pectins are de-esterified by pectin methyl-esterase (PME) to be acid pectins [45]. It was found that the negatively charged carboxyl groups in pectin combined with Al^3+^ in large quantities, which reduced the toxicity of Al^3+^ to plant cells [41]. The amount of negatively-charged carboxyl groups in pectin is determined by its de-methyl esterification degree, which is controlled by the activity of PME [46]. It was found that Al-tolerant varieties showed higher esterified pectin ratios and lower PME activity in rice, corn, and buckwheat [47]. The activity of PME in root tips was negatively correlated with the degree of pectin methylation and positively correlated with the accumulation of Al [48]. Decreased acid pectins at the tip could result in a more intense action of the turgor pressure and therefore a faster growth of pollen tubes.

In our previous research, Fang et al. (2020) reported that 600 μM AlCl_3_ increased acid pectins and had no obvious effects on esterified pectins at apple the pollen tube tip [1]. After 600 μM AlCl_3_ treatment, very weak fluorescence of (1→5)-α-L-arabinan was detected at the pollen tube wall [1].

In this study, 20 µM AlCl_3_ treatment decreased acid pectin but increased esterified pectin in the walls of apple pollen tubes. In conclusion, 20 µM AlCl_3_ could stimulate apple pollen germination and pollen tube growth by enhancing Ca^2+^ influx and altering cell wall components.

## 4. Materials and Methods

### 4.1. Plant Materials and Pollen Culture

The material used in this experiment was apple pollen grain, which was collected at maturity in Shandong Province, China, on 12 April 2019. The collected mature pollen grain was evenly spread on sulfate paper. After drying, the pollen grain was collected in 50 mL centrifuge tubes, sealed in darkness, and stored at −20 °C for later use.

Totals of 0, 20 µM, and 50 µM AlCl_3_ (Sigma, 563919, Oakville, CA, USA) were added to the basic liquid medium, which consisted of 20% (*w/v*) sucrose (Sigma, V900116), 0.015% CaCl_2_ (Sigma, C1016) and 0.01% H_3_BO_3_ (Sigma, B0394). Pollen grain was cultured in the above media in darkness at 30 °C.

After being incubated for 2 h, pollen germination rate was calculated under a BX51 microscope (Olympus, Tokyo, Japan) [49]. MetaMorph (Universal Imaging) was employed to measure the length of the pollen tubes [49]. A total of 150 pollen grains were counted in each experiment (repeated three times) of pollen germination rate and pollen tube length. Statistical tests were employed by SPSS.

### 4.2. Measurement of Extracellular Ca^2+^ Flux

The Net Ca^2+^ flux at the tip of pollen tubes was measured using a Non-invasive Micro-test Technique (BIO-001B, Younger USA Sci. and Tech. Corp., Amherst, MA, USA) according to [49]. We calculated the Ca^2+^ fluxes using Mageflux software (V 3.0). The experiments were repeated three times in each group, and the samples were measured for 10 min each time.

### 4.3. Fluorescence Labeling of Pollen Tube Cytoplasmic [Ca^2+^]c

According to [49], the [Ca^2+^]c of pollen tubes was labeled with Fluo-3/AM ester (final concentration 100 μM, Sigma-Aldrich) for 1 h at 4 °C in darkness. Then, the pollen tubes were washed with the culture medium three times. After that, the pollen tubes were placed at room temperature for 1 h. The pollen tubes were then observed, and images were captured using a confocal laser scanning microscope (CLSM, Leica TCS SP5, Mannheim, Germany) with excitation wavelength 488 nm and emission wavelength 510–530 nm.

### 4.4. Fluorescence Labeling of Actin Filaments

Actin filaments were labeled using the methods described in [49]. In short, pollen tubes were fixed with 4% paraformaldehyde for 1.5 h, followed by three washes with PBS. Then, the pollen tubes were treated with 1% pectinase and 1% cellulase for 15 min. After that, the pollen tubes were treated with 1% Triton for 1 h. Finally, the pollen tubes were labeled with 0.2 μM phalloidin-FITC for 2 h in darkness. The actin filaments of the pollen tubes were observed under CLSM with excitation wavelength 488 nm and emission wavelength 510–530 nm.

### 4.5. Fluorescence Localization and Analysis of Pollen Tube Wall Components

According to [49], Calcofluor White Stain and aniline blue were employed to label cellulose and callose, respectively. Then, an ultraviolet channel (excitation filter BP395-440; chromatic beam splitter FT460; barrier filter LP470) was used to observe the fluorescence of cellulose and callose under a fluorescence microscope (Olympus BX51, Tokyo, Japan).

Monoclonal antibodies JIM5, JIM7, LM6 (University of Leeds, UK; diluted at 1:10) and the secondary antibody FITC-labeled sheep anti-rat IgG (Sigma-Aldrich; diluted at 1:100 in PBS) were selected to label acid pectins, esterified pectins, and arabinan pectins, respectively. CLSM was used to observe the fluorescence of pectins with excitation wavelength 488 nm and emission wavelength 510–530 nm. Controls were set by omitting the primary antibody.

Quantification of fluorescent intensity was performed according to [50] using ImageJ software (Rasband, W.S., ImageJ, U. S. National Institutes of Health, Bethesda, ML, USA, http://rsb.info.nih.gov/ij/, 1997–2012 (accessed on 1 January 2006)). Pixel intensity was measured along the periphery of the pollen tubes, beginning from the tip (the outermost tip of the tube). Values on the x-axes in the graphs indicate the meridional distance from the tip of the pollen tube. Ten pollen tubes were selected and analyzed for each treatment at random. Three independent experiments were conducted for each measurement.

In our research, all the conditions were the same when images were taken. Even the experimental conditions (including the time, frequency of pollen tube cleaning, labeling, and so on) were the same. So, all the images have a common background.

The chemical composition of pollen tube tip wall was analyzed using Fourier Transform Infrared (FTIR) spectroscopy according to [50]. For each treatment, ten pollen tubes were analyzed randomly, and three repetitions were conducted.

## Figures and Tables

**Table 1:** Germination rate and length of pollen tubes under different concentrations of AlCl_3_.

Concentration of AlCl_3_/µM	Germination Rate/%	Pollen Tube Length/μm
0	35.09 ± 2.33 ^cd^	101.83 ± 8.18 ^c^
10	42.07 ± 1.90 ^bc^	138.46 ± 8.92 ^b^
20	57.25 ± 3.29 ^a^	142.36 ± 4.80 ^ab^
30	42.88 ± 1.72 ^b^	141.88 ± 7.21 ^ab^
50	33.89 ± 2.98 ^d^	144.64 ± 10.47 ^ab^
100	31.97 ± 1.37 ^d^	147.43 ± 7.85 ^a^

Note: Different letters indicated statistically significant differences between pollen tubes grown at various conditions (*p* ≤ 0.05).

## Data Availability

Not applicable.

## References

[1] Fang K.F., Xie P.X., Zhang Q., Xing Y., Cao Q.Q., Qin L. (2020). Aluminum toxicity-induced pollen tube growth inhibition in apple is mediated by interrupting calcium dynamics and modification of cell wall components. Environ. Exp. Bot..

[2] Ranjan A., Sinha R., Sharma T.R., Pattanayak A., Singh A.K. (2021). Alleviating aluminum toxicity in plants: Implications of reactive oxygen species signaling and crosstalk with other signaling pathways. Plant Physiol..

[3] Osaki M.T., Watanabe T., Tadano T. (1997). Beneficial effect of aluminum on growth of plants adapted to low pH soils. Soil Sci. Plant Nutr..

[4] Watanabe T., Jansen S., Osaki M. (2005). The beneficial effect of aluminium and the role of citrate in Al accumulation in *Melastoma malabathricum*. New Phytol..

[5] Du B., Nian H., Zhang Z., Yang C. (2010). Effects of aluminum on superoxide dismutase and peroxidase activities, and lipid peroxidation in the roots and calluses of soybeans differing in aluminum tolerance. Acta Physiol Plant..

[6] Wang L., Fan X.W., Pan J.L., Huang Z.B., Li Y.Z. (2015). Physiological characterization of maize tolerance to low dose of aluminum, highlighted by promoted leaf growth. Planta.

[7] Pilon-Smits E.A., Quinn C.F., Tapken W., Malagoli M., Schiavon M. (2009). Physiological functions of beneficial elements. Curr. Opin. Plant Biol..

[8] Zhu X., Tang C., Li Q., Qiao X., Li X., Cai Y., Wang P., Sun Y., Zhang H., Zhang S. (2021). Characterization of the pectin methylesterase inhibitor gene family in Rosaceae and role of *PbrPMEI23/39/41* in methylesterified pectin distribution in pear pollen tube. Planta.

[9] Hepler P.K., Winship L.J. (2015). The pollen tube clear zone: Clues to the mechanism of polarized growth. J. Integr. Plant Biol..

[10] Bou D.F., Geitmann A. (2011). Actin is involved in pollen tube tropism through redefining the spatial targeting of secretory vesicles. Traffic.

[11] Zhang Y., He J., Lee D., McCormick S. (2010). Interdependence of endomembrane trafficking and actin dynamics during polarized growth of *Arabidopsis* pollen tubes. Plant Physiol..

[12] Zhou Y.N., Cui X.Y., Hu A.N., Miao Y.H., Zhang L.Y. (2020). Characterization and functional analysis of pollen-specific *Pw SWEET1* in *Picea wilsonii*. J. Forestry Res..

[13] Zonia L., Munnik T. (2011). Understanding pollen tube growth: The hydrodynamic model versus the cell wall model. Trends Plant Sci..

[14] Xie B., Deng Y., Kanaoka M.M., Okada K., Hong Z. (2012). Expression of *Arabidopsis* callose synthase 5 results in callose accumulation and cell wall permeability alteration. Plant Sci..

[15] Hepler P.K., Winship L.J. (2010). Calcium at the cell wall-cytoplast interface. J. Integr. Plant Biol..

[16] Sawidis T., Reiss H.D. (1995). Effects of heavy metals on pollen tube growth and ultrastructure. Protoplasma.

[17] Sade H., Meriga B., Surapu V., Gadi J., Sunita M.S., Suravajhala P., Kavi Kishor P.B. (2016). Toxicity and tolerance of aluminum in plants: Tailoring plants to suit to acid soils. Biometals.

[18] Zhang W.H., Rengel Z., Kuo J., Yan G. (1999). Aluminium effects on pollen germination and tube growth of *Chamelaucium uncinatum*. A comparison with other Ca^2+^ antagonists. Ann. Bot..

[19] Konishi S., Miyamoto S. (1983). Alleviation of aluminum stress and stimulation of tea pollen tube growth by fluorine. Plant Cell Physiol..

[20] Hajiboland R., Barceló J., Poschenrieder C., Tolrà R. (2013). Amelioration of iron toxicity: A mechanism for aluminum-induced growth stimulation in tea plants. J. Inorg. Biochem..

[21] Hiromi Y., Ikuyo T., Fumiko I., Miwako O., Shigeki K. (1997). Stimulatory effect of aluminum on the growth of tea pollen tubes. Soil Sci. Plant Nutr..

[22] Mollet J.C., Leroux C., Dardelle F., Lehner A. (2013). Cell wall composition, biosynthesis and remodeling during pollen tube growth. Plants.

[23] Shetty R., Vidya C.S., Prakash N.B., Lux A., Vaculík M. (2021). Aluminum toxicity in plants and its possible mitigation in acid soils by biochar: A review. Sci. Total Environ..

[24] Le Poder L., Mercier C., Février L., Duong N., David P., Pluchon S., Nussaume L., Desnos T. (2022). Uncoupling Aluminum toxicity from Aluminum signals in the *STOP1* pathway. Front. Plant Sci..

[25] Bushart T.J., Roux S.J. (2007). Conserved features of germination and polarized cell growth: A few insights from a pollen-fern spore comparison. Ann Bot..

[26] Miller D.D., Callaham D.A., Gross D.J., Hepler P.K. (1992). Free Ca^2+^ gradient in growing pollen tubes of *lilium*. J. Cell Sci..

[27] Monshausen G.B., Messerli M.A., Gilroy S. (2008). Imaging of the Yellow Cameleon 3.6 indicator reveals that elevations in cytosolic Ca^2+^ follow oscillating increases in growth in root hairs of *Arabidopsis*. Plant Physiol..

[28] Huang J.W., Shaff J.E., Grunes D.L., Kochian L.V. (1992). Aluminum effects on calcium fluxes at root apex of aluminum-tolerant and aluminum-sensitive wheat cultivars. Plant Physiol..

[29] Rengel Z., Zhang Z.H. (2003). Role of dynamic of intracellular calcium in aluminum toxicity syndrome. New Phytol..

[30] Very A.A., Davies J.M. (2000). Hyperpolarization-activated calcium channels at the tip of *Arabidopsis* root hairs. Proc. Natl. Acad. Sci. USA.

[31] Rengel Z. (1992). Role of calcium in aluminum toxicity. New Phytol..

[32] Hepler P.K. (2005). Calcium: A central regulator of plant growth and development. Plant Cell.

[33] Kopittke P.M., Moore K.L., Lombi E., Gianoncelli A., Ferguson B.J., Blamey F.P.C., Menzies N.W., Nicholson T.M., McKenna B.A., Wang P. (2015). Identification of the primary lesion of toxic aluminum in plant roots. Plant Physiol..

[34] Baldwin L., Domon J.M., Flimek J.F., Fournet F., Sellier H., Gillet F., Pelloux J., Lejeune-Hénaut I., Carpita N.C., Rayon C. (2014). Structural alteration of cell wall pectins accompanies pea development in response to cold. Phytochemistry.

[35] Sun P., Tian Q.Y., Zhao M.G. (2007). Aluminum-induced ethylene production is associated with inhibition of root elongation in *Lotus japonicus* L.. Plant Cell Physiol..

[36] Chen X.Y., Kim J.Y. (2009). Callose synthesis in higher plants. Plant Signal Behav..

[37] Allison A.V. (1974). The ultrastructure of local lesions induced by potato virus x: A sequence of cytological events in the course of infection. Phytopathology.

[38] Shi X., Han X., Lu T.G. (2016). Callose synthesis during reproductive development in monocotyledonous and dicotyledonous plants. Plant Signal Behav..

[39] Parre E., Geitmann A. (2005). More than a leak sealant. The mechanical properties of callose in pollen tubes. Plant Physiol..

[40] Mohnen D. (2008). Pectin structure and biosynthesis. Curr. Opin. Plant Biol..

[41] Nagayama T., Nakamura A., Yamaji N., Satoh S., Furukawa J., Iwai H. (2019). Changes in the distribution of pectin in root border cells under aluminum stress. Front. Plant Sci..

[42] Yang J.L., Li Y.Y., Zhang Y.J., Zhang S.S., Wu Y.R., Wu P., Zheng S.J. (2008). Cell wall polysaccharides are specifically involved in the exclusion of aluminum from the rice root tip. Plant Physiol..

[43] Hasegawa Y., Nakamura S., Kakizoe S., Sato M., Nakamura N. (1998). Immunocytochemical and chemical analyses of Golgi vesicles isolated from the germinated pollen of *Camellia japonica*. J. Plant Res..

[44] Franklin-Tong V.E. (1999). Signaling and the modulation of pollen tube growth. Plant Cell.

[45] Geitmann A., Cresti M., Cai G., Moscatelli A. (1999). The rheological properties of the pollen tube cell wall. Fertilization in Higher Plants.

[46] Wu Q., Tao Y., Zhang X.L., Dong X.Y., Xia J.X., Shen R.F., Zhu X.F. (2022). Pectin methylesterases enhance root cell wall phosphorus remobilization in rice. Rice Sci..

[47] Wei Y.M., Han R.R., Xie Y.H., Jiang C.D., Yu Y.X. (2021). Recent advances in understanding mechanisms of plant tolerance and response to aluminum toxicity. Sustainability.

[48] Yang X.Y., Zeng Z.H., Yan J.Y., Fan W., Bian H.W., Zhu M.Y., Yang J.L., Zheng S.J. (2013). Association of specific pectin methylesterases with Al-induced root elongation inhibition in rice. Plant Physiol..

[49] Fang K.F., Zhang W.W., Xing Y., Zhang Q., Yang L., Cao Q.Q., Qin L. (2016). Boron toxicity causes multiple effects on *Malus Domestica* pollen tube growth. Front. Plant. Sci..

[50] Chebli Y., Pujol L., Shojaeifard A., Brouwer I., van Loon J.J., Geitmann A. (2013). Cell wall assembly and intracellular trafficking in plant cells are directly affected by changes in the magnitude of gravitational acceleration. PLoS ONE.

